# Features of sacral alar fatigue fractures in adolescent athletes with overuse

**DOI:** 10.1038/s41598-021-87752-4

**Published:** 2021-04-19

**Authors:** Masaki Tatsumura, Fumihiko Eto, Katsuya Nagashima, Shun Okuwaki, Hisanori Gamada, Sho Iwabuchi, Takeshi Ogawa, Takeo Mammoto, Atsushi Hirano, Masashi Yamazaki

**Affiliations:** 1 Department of Orthopaedic Surgery and Sports Medicine, Tsukuba University Hospital Mito Clinical Education and Training Center, Mito Kyodo General Hospital, 3-2-7 Miyamachi, Mito, Ibaraki 310-0015 Japan; 2 Department of Orthopaedic Surgery, Faculty of Medicine, University of Tsukuba, Tsukuba, Japan

**Keywords:** Anatomy, Medical research

## Abstract

Three types of sacral alar fatigue fractures are elderly, postnatal, and sport-related. They are most prevalent in athletes during adulthood; there are few reports of sacral alar fatigue fractures in young athletes. The purpose of this study was to analyze sacral alar fatigue fractures in adolescent athletes. Of the 920 patients hospitalized with low back pain, 13 were diagnosed with sacral alar fatigue fractures with magnetic resonance imaging (MRI) abnormalities. We investigated age, sex, sports discipline, span from symptom onset to consultation, laterality, complication with spondylolysis, computed tomography (CT) findings, and treatment span. The average age was 14.5 years old (8-men and 5-women). The most frequent discipline was basketball. The span to consultation was 13.2 days. The number of right-side cases was 9. Seven cases were complicated by bilateral spondylolysis. MRI abnormalities were observed in all the cases. Only two patients showed abnormal findings on CT. Averagely 67 days after treatment, participants returned to their sports. Sacral alar fatigue fractures suggest that the span from onset to consultation is short. Fracture lines are often unclear on CT, and MRI is useful for diagnosis. More than half of the cases in this study were complicated by lumbar spondylolysis.

## Introduction

Unlike older people, adolescent athletes have a lower incidence of nonspecific degenerative changes. Therefore, fatigue fractures of the lumbar spine or sacrum are one of the causes of sports-related lower back pain in adolescents. Kaneko et al. reported that 33.0% of the causes of low back pain in athletes under 18 years old were lumbar fatigue fractures^1^. In that report, sacral stress fractures accounted for only 1.6% of patients with low back pain. Approximately half of the adolescent athletes who come to our hospital with lumbago as the chief complaint have lumbar spondylolysis^2^. However, fatigue fractures of the sacral alar are exceedingly rare^3^.

Some sacral alar fatigue fractures are due to bone fragility in the elderly^4^ or in postpartum women^5^, but they are rarely due to overexercise in younger age groups. There are a few case reports, in which it is considered that the frequency of sport related sacral alar fatigue fractures in adolescent is not significantly high. Sacral fatigue fractures due to overexercise in adults have been reported as case series^6^. However, adolescent cases are even rarer. In this study, we examined the features of sacral alar fatigue fractures in 13 adolescent patients.

## Methods

### Patients

Of the 920 high school students and younger children who visited our hospital with back pain as the chief complaint in the duration of 6 years from April 2014, 13 cases were diagnosed with sacral alar fatigue fracture. We detected sacral alar fatigue fracture using short tau inversion recovery (STIR) and magnetic resonance imaging (MRI).

### Methods

We analyzed age, sex, sports disciplines, laterality, and the duration from the onset date to the hospital visit. Additionally, we investigated the existing number of abnormal findings on computed tomography (CT), the area of bone marrow edema on MRI, complicated cases with spondylolysis, and treatment duration. Conservative treatments include rest from sporting activities, physical therapy, and wearing a brace. Regarding physical therapy, while the patient was in pain, medical rehabilitation was performed using isometric exercises of the trunk, whereas athletic rehabilitation with dynamic exercises was performed after the pain disappeared. Pain killers were not used because the resting pain was mild in all cases. The end point of conservative treatment was the day on which we confirmed the diminishing of signal change on MRI. MRI was performed monthly, and two orthopedic surgeons performed the interpretation. By comparing the signal intensity on the healthy side, we judged the diminishing signal change of the lesion. This research was approved by the IRB of Tsukuba University Hospital Mito Clinical Education and Training Center/ Mito Kyodo General Hospital, and the methods in this study were performed according to the guidelines and regulations. Written informed consent was obtained from the patients’ parents or legal guardians.

### Ethics approval

All procedures including review of patient record used in this research were approved by the institutional review board.

## Results

**Figure 1:**
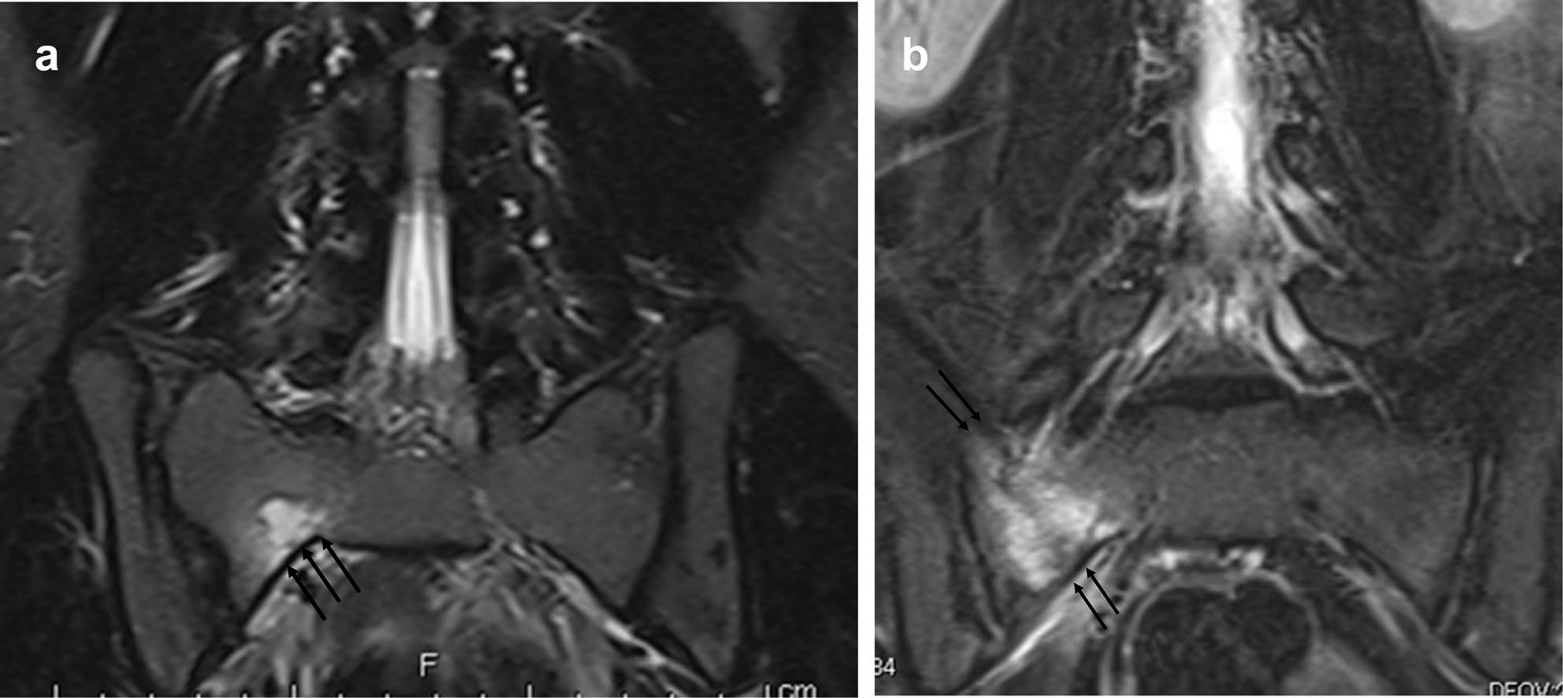
Characteristic of the area of bone marrow edema on MRI STIR. (**a**) This is a sample view of bone marrow edema that existed locally around the superior sacral foramen on the MRI STIR coronal view (arrows indicate bone marrow edema). (**b**) This is a sample view of bone marrow edema extending from the superior anterior sacral foramina to the superior lateral sacral alar parallel to the sacroiliac joint on the MRI STIR coronal view (arrows indicate bone marrow edema).

The average age of the study population was 14.5 years (11–18 years), and the number of patients with respect to sex was 8 young men and 5 young women. The sports disciplines were basketball (6 cases), baseball (2 cases), soccer (1 case), lacrosse (1 case), distance running (1 case), wrestling (1 case), and volleyball (1 case). Patient demographics are shown in Table 1. There were 9 cases with fractures observed on the right side and 4 cases on the left side. The average duration from the appearance of symptoms to the hospital visit was 13.2 days (1–31 days). Abnormal findings on CT revealed a visible fracture line in one patient and a sclerotic change in one patient. Regarding the area of bone marrow edema on MRI, bone marrow edema existed locally around the superior sacral foramen in 8 cases (Fig. 1a), extending from the superior anterior sacral foramina to the superior lateral sacral alar parallel to the sacroiliac joint in 5 cases (Fig. 1b). Six of the 13 patients had no lumbar spine disease, but 7 patients had lumbar spondylolysis at the time of diagnosis of sacral alar fatigue fracture. Three cases of lumbar spondylolysis were in the acute phase with bone marrow edema on the pars interarticularis, and the remaining four cases were in the chronic phase with pseudarthrosis. The treatment span was 67 days (range, 33–101 days), and the back pain improved in all cases.

**Table 1:** Patients’ demographic data. Sex, age at the first visit, sports discipline, duration from symptoms to visit, laterality, abnormal findings on CT, the area of bone marrow edema on MRI, and complicated cases with spondylolysis.

Case	Age	Sex	Sport discipline	Days from symptoms to visit	Laterality	Abnormality on CT	Area of MRI Change	With Spondylolysis
1	15	M	Basketball	8 days	Right	No Change	Local Change	(−)
2	11	F	Basketball	10 days	Left	No Change	Extended Change	(+)
3	11	F	Basketball	7 days	Right	Fracture Line	Extended Change	(−)
4	15	M	Volleyball	31 days	Left	No Change	Local Change	(−)
5	15	F	Basketball	1 days	Right	No Change	Local Change	(−)
6	18	F	Basketball	6 days	Right	No Change	Extended Change	(+)
7	17	M	Soccer	5 days	Right	No Change	Local Change	(−)
8	18	F	Lacross	14 days	Right	No Change	Local Change	(−)
9	11	M	Baseball	20 days	Right	No Change	Local Change	(+)
10	16	M	Basketball	31 days	Left	Sclerotic Change	Extended Change	(+)
11	12	M	Wrestling	12 day	Right	No Change	Extended Change	(+)
12	16	M	Distance Running	13 days	Right	No Change	Local Change	(+)
13	14	M	Baseball	14 days	Left	No Change	Local Change	(+)

## Representative case

**Figure 2:**
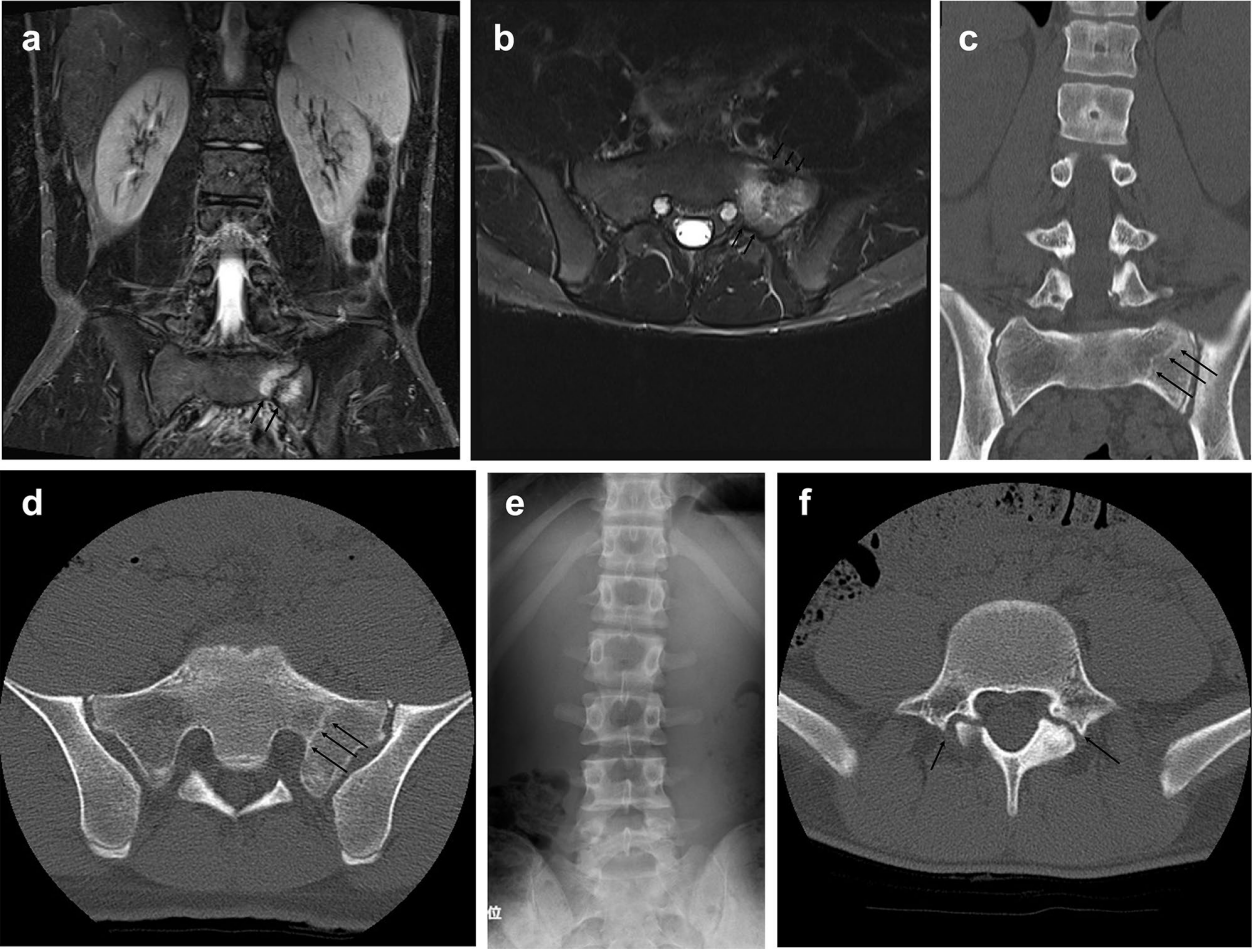
Pretreatment images of the representative case. (**a**) The MRI STIR coronal view shows a signal changed on the oblique line on the left sacral alar (arrows indicate a signal changed line). (**b**) The MRI STIR axial view shows a massive change in the signal on the left sacral alar (arrows indicate bone marrow edema). (**c**) CT coronal view showing a sclerotic oblique line on the left sacral alar (arrows indicate sclerotic line). (**d**) CT axial view of the sacrum shows a sclerotic oblique line on the left sacral alar (arrows indicate sclerotic line). (**e**) Plain radiograph AP view shows no abnormal changes. (**f**) CT axial view of the fifth lumbar shows pseudarthrotic bilateral spondylolysis (arrows indicate bilateral bony cleft).

**Figure 3:**
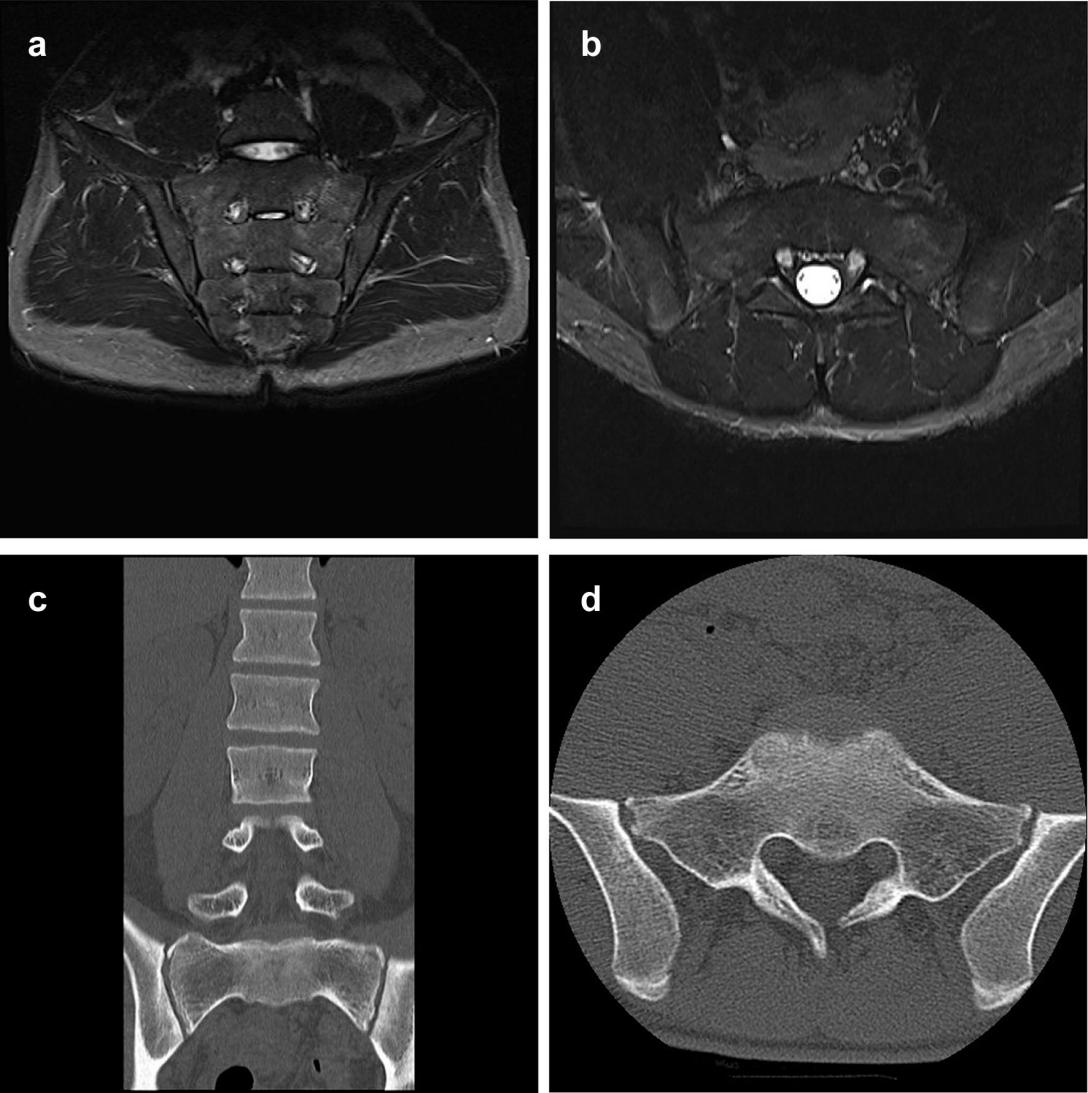
Images after treatment of the representative case. (**a**) MRI STIR coronal view shows a normal signal on the left sacral alar. (**b**) MRI STIR axial view shows a normal signal on the left sacral alar. (**c**) CT coronal view shows no fracture line on the left sacral alar. (**d**) CT axial view of the sacrum showed no fracture line on the left sacral alar.

A 16-year-old boy with a history of pseudarthrotic lumbar spondylolysis presented to our hospital complaining of low back pain since 13 days. Physical examination revealed low back pain during trunk extension and restricted range of motion for extension. No symptoms were observed during flexion or in the sciatic nerve. During this visit, the MRI STIR showed bone marrow edema on the right side of the sacral alar (Fig. 2a–b). CT showed a slightly sclerotic line at the right sacral alar (Fig. 2c–d). However, plain radiography showed no abnormal changes (Fig. 2e). He had a history of lumbar spondylolysis, and CT imaging showed pseudarthrotic spondylolysis of L5 (Fig. 2f). Conservative therapy with rest and the use of a soft brace was prescribed. He also participated in physical therapy at least once per week. Forty-four days after the first visit, MRI STIR showed a normal signal, indicating that the lesion had resolved (Fig. 3a–b), and CT revealed no abnormality (Fig. 3c–d). After confirmation of recovery, the patient returned to play basketball.

## Discussion

Non-traumatic sacral alar fractures are roughly divided into three categories: fragile fractures due to decreased bone density in the elderly, postpartum fractures due to bone metabolic changes in women, and fatigue fractures due to overuse in athletes. Young athletes’ sacral fatigue fractures were first reported in 1984^7^ and were most common in zone 1 of the Denis classification and are often accompanied by a fracture line parallel to the sacroiliac joint^8^. Among athletes, many reports about fatigue fracture of the sacral alar have been reported in adults^6^; however, the cases in adolescents are rarely reported. Since adolescents have developing bones with high elasticity, sport-related fatigue fractures are likely to occur. Sacral alar fatigue fracture and lumbar spondylolysis in adolescents are more likely to occur in athletes because they are caused by excessive repetitive movements of the trunk.

The mechanism of sacral alar fatigue fractures is that the axial stress transmitted from the lordotic lumbar spine transforms into shear force on the sacrum because the sacrum is inclined with respect to the horizontal plane^9^. This is due to the shear force applied to the sacrum from the vertebral bodies during running and jumping movements. As for the five cases of Kaneko et al., two of these patients were baseball players, two were soccer players, and one was a basketball player^1^. Basketball players were the dominant players in this study. It seems that the external force on the sacrum by jumping and landing impaction was the main cause of the fractures.

We encountered 13 cases of a sacral alar fatigue fracture in adolescents. The prevalence of sacral alar fatigue fracture in this study was 1.4% in patients who had back pain and was similar to the study by Kaneko et al., in which 1.6% of patients experienced sacral alar fatigue fracture^1^. The average age of this study population was 14.5 years, which was similar to the study in lumbar spondylolysis treated at our hospital, in which the average age was 14.7 years old^2^. The ratio of young men to women was 8:5, which was similar to the ratio of lumbar spondylolysis in this study. It has been reported that sacral alar fatigue fractures are more common among women because they are associated with female athlete triad^10^. However, it was observed to be male-dominated with adolescents in this study.

The duration from the onset date to undergoing medical examination was only 13.2 days. It is speculated that pain of sacral alar fatigue fractures is more serious. Because the pain of sacral alar fatigue fractures may be stronger than pain of lumbar spondylolysis, the patient is seen earlier from the onset to relief their pain.

In this series, there were 9 cases in which the fracture occurred on the right side, making fractures more common on the right side than on the left. There were differences in the laterality of lumbar spondylolysis because the frequencies on the left or right sides may be different because of the repetition of asymmetrical movements based on the discipline of the sport^11^. It is thought that sports-related asymmetrical movements also cause laterality in sacral alar fatigue fractures.

Despite a case report of bilateral sacral alar fatigue fracture^12^, no bilateral case was reported in this study. Fractures on the opposite side have been reported to arise easily when one side fail to fuse^13^ and fracture with contralateral bony cleft will easily fail to fuse in lumbar spondylolysis^14^. However, sacral alar fatigue fractures have a high rate of fusion because fractures on both sides are unlikely to occur simultaneously. In addition, since it is painful and difficult to continue sports, it is presumed that the doctor will often see the patient with a fracture on one side before fractures occur on both sides.

Although it is often difficult to make a diagnosis using plain radiography or CT, bone marrow edema on sacrum could be visualized clearly on MRI STIR. Therefore, an MRI is the first choice for diagnostic purposes^15^ because of its high sensitivity^6^. The characteristic feature of MRI was the initial appearance of the bone marrow around the cranial dorsal side of the sacral foramen. As the fracture progressed, the bone marrow edema extended, and a fracture line was recognized toward the superior parallel to the sacroiliac joint on MRI. CT showed that the fracture line was parallel to the sacroiliac joint in only one case in this study. In some cases, linear sclerosis with cortical disruption can be observed on CT^16^. We could not see any abnormality in the majority of the cases. Therefore, CT is unsuitable for identification of fractures with respect to lumbar spondylolysis in the early phase. Thus, MRI is necessary to diagnose it even in the absence of findings on CT.

Usually, most adolescent athletes who complain of low back pain during extension are observed to have lumbar spondylolysis. Therefore, if an imaging examination is performed only for lumbar spondylolysis, the sacrum may be outside the imaging range. The diagnosis of sacral alar fatigue fractures was reported to improve upon the addition of a pelvic coronal view^17^. The STIR condition of the coronal view of the sacrum is essential for the diagnosis of low back pain in junior or senior high school students. It was able to identify signal changes in the sacral alar in the MRI images of patients with suspected lumbar spondylolysis because the upper sacrum is often included in the imaging range even when imaging is performed with lumbar coils. In particular, bone marrow edema was clearer in the coronal view. For young athletes who complain of low back pain, the coronal view should be included in the conditions for MRI, and sacral alar fractures should be considered and evaluated along with the lumbar spine. Care must be taken not to miss it because it can be visualized in the coronal view, even when imaging with the lumbar coil.

Sacral alar fatigue fractures complicated radicular pain in a few cases^16^. In all the cases in this study, signal changes around the anterior sacral foramen were observed. However, no radiculopathy appeared. The main clinical symptoms of both sacral alar fatigue fracture and lumbar spondylolysis are low back pain during movement. Hence, the differential diagnosis of lumbar spondylolysis seems to be difficult on physical examination. Thus, an imaging examination must be performed for an accurate diagnosis.

In the case of therapeutic intervention, the pain disappeared, and it was possible to return to sporting activities after exercise inhibition and physical therapy. The treatment span was approximately 67 days, which was shorter than that for lumbar spondylolysis. However, there was only one case of contralateral occurrence 6 months after returning to the competition. Therefore, training after recovery should be performed carefully. Compared with sacral stress fractures, lumbar spondylolysis is an intractable disease with a very low union rate of 76% and a treatment period of 106 days^18^. Nonetheless, we believe that differential diagnosis is important to give patients peace of mind.

There have been case reports of sacral alar fatigue fractures occurring after the treatment of lumbar spondylolysis^19^. Notably, more than half of the cases in this study were found to have complications with spondylolysis.

## Conclusion

Lower back pain in developing athletes includes sacral alar fatigue fractures with a frequency of 1.4%. MRI is useful because it is difficult to diagnose fractures based on clinical findings and CT. Additionally, sacral alar fatigue fractures should be considered. During evaluation, it is necessary for the sacral alar fractures to also be considered.

### Consent to participate

Oral informed consent was obtained from all patients and their parents to participate in this study.

## Data Availability

The datasets generated during and analyzed during the current study are available from the corresponding author on reasonable request.
